# The Exponential Rise of Teledentistry and Patient-Oriented Protective Measures in Southeast Asian Dental Clinics: Concerns, Benefits, and Challenges

**DOI:** 10.1155/2021/9963329

**Published:** 2021-10-06

**Authors:** Anand Marya, Adith Venugopal, Mohmed Isaqali Karobari, Pietro Messina, Giuseppe Alessandro Scardina, Aravind Kumar Subramanian

**Affiliations:** ^1^Department of Orthodontics, Faculty of Dentistry, University of Puthisastra, Phnom Penh, Cambodia; ^2^Department of Orthodontics, Saveetha Dental College, Saveetha Institute of Medical and Technical Sciences, Saveetha University, Chennai, India; ^3^Conservative Dentistry Unit, School of Dental Sciences, Universiti Sains Malaysia, Health Campus,16150 Kubang Kerian, Kota Bharu, Kelantan, Malaysia; ^4^Department of Surgical,Oncological and Stomatological Disciplines, University of Palermo, Palermo, Italy

## Abstract

In the Southeast Asian region, various policies have been advocated by health regulatory bodies that entail protective measures such as face masks, gloves, maintaining distance in public areas, and more. These protective measures are aimed at helping reverse the growth rate of the coronavirus. Dentists in this region have incorporated several changes to their practices to help minimize risks of person-to-person transmission inside dental offices. This narrative review aimed to provide an in-depth overview of the current situation in the Southeast Asian region regarding the use of teledentistry during the pandemic. Teledentistry involves the transfer of patient information across remote distances for online consultation and treatment planning. A few years back, it used to be a lesser-known entity but has seen an exponential rise in its incorporation into dental practices all around the Association of Southeast Nations (ASEAN) region. Many clinics in the Southeast Asian region have started using online consultations to ensure that patients can be diagnosed or followed up during their treatment. Teledentistry is the clear answer in the coming months as it will help reduce the risk of virus transmission and help patients get access to oral healthcare and dentists to see their patients. This article reviews the current pandemic situation in the ASEAN region, the recent evidence, and the scope of teledentistry. It also provides recommendations for the future and sheds light on the different types of teledentistry and how it can be incorporated into practices by regulatory authorities in this region.

## 1. Introduction

At the time of preparing this review, the entire world has been affected by the COVID-19 pandemic. While the Southeast Asian region has seen a lesser incidence of COVID-19 than the developing countries, many countries have gone into lockdown again in the past few months because of rising new cases [1]. Several measures have been implemented in this region to aid in the containment of the virus, including lockdowns, travel restrictions across borders, and the advocacy of different safety protocols depending on the geographical location. In the Southeast Asian region, various policies have been advocated by health regulatory bodies that entail measures such as the use of protective face masks and gloves and maintaining distance in public areas, all of which are aimed at helping to reverse the growth rate of the virus [2]. Among these measures, the use of virtual dentistry, or teledentistry as we call it, has been rising during this period. The Southeast Asian region, as we know, includes three groups of countries: the lower-middle-income, upper-middle-income, and the high-income group of countries. The lower-middle-income group is formed by Indonesia, Cambodia, Sri Lanka, the Philippines, and Vietnam. The upper-middle-income group comprises Malaysia and Thailand, while Singapore has been placed in the high-income group of countries (Tables 1 and 2) [3, 4].

One of the principal problems in this region is oral health, and there have been no cross-national studies conducted to evaluate the oral health status. National oral healthcare plans have not been organized and implemented in many countries in this region due to budgetary restrictions. There are very less public oral health services because of which dental problems are widely prevalent here. It has been shown in previous studies conducted in the Association of Southeast Nations (ASEAN) region that people who received treatment for severe dental problems were forced to reduce expenses on necessities because of the additional costs incurred [5–12]. Teledentistry is a cost-effective way to deliver primary as well as specialty care [13, 14]. The problem is that, before the pandemic, teledentistry was considered an optional form of oral healthcare delivery rather than a mainstream one [15]. There have been several challenges in implementing virtual dentistry in Southeast Asia and globally, such as technical issues, expenditure, Internet speed, political clearances, and health infrastructure [16–18].

Teledentistry has been defined as the virtual delivery of dental services by various means such as video, audio, or multimedia [19, 20]. These dental services can include gathering records for diagnosis, consultations, and treatment follow-up using a systematic collection of records [21]. The primary benefit of this technology is that it can benefit patients based in remote areas to get access to healthcare without physically covering the distance to see their dentist [22]. Many clinics in the Southeast Asian region have started using this virtual modality to ensure that patients can be diagnosed or followed up during their treatment.

## 2. Brief Review of the Literature

Over the past few years, Teledentistry has been proven to be highly effective in disseminating access and advantageous in terms of a profound reduction in the treatment time and costs [23]. Previous studies have analyzed the cost-benefit ratio across patients located in rural areas and found teledentistry to be a financially cost-effective option. Virtual dentistry has also been demonstrated to be helpful when it comes to remote screening for oral lesions, oral health education programs, and virtual consultations in remote rural areas [24]. In Latin America, Information and Communication Technologies (ICT) is considered a part of dental health provision and has been highly efficient in educational programs and research [25, 26]. The major problem faced by developing countries in using teledentistry has been attributed to many factors such as shortage of infrastructure, insufficient resources, materials, and even the conservative thought process of the governing bodies [27]. During the past year, many concerns have arisen regarding the risk of cross infection from patients visiting dental clinics for treatment. Governing bodies have set down various guidelines to prevent the spread of the SARS-CoV-2 virus [28–30]. Some of the recommendations include patient screening at the clinic entrance, telephonic screening before the patient visit, recording the patient's recent travel history to infection hotspots, and even the use of virtual consultations to alleviate patient concerns [31].

## 3. Patient-Oriented Changes Incorporated by Dental Clinics in the ASEAN Region

Dentists in this region have incorporated several changes to their practices to help minimize risks of person-to-person transmission inside dental offices [32]. Staff-oriented measures such as thermal screening at the clinic entry, hand hygiene utilizing handwashes and alcohol before wearing gloves, and the diligent use of personal protective equipment have been implemented [33]. Patients have been asked to complete additional travel and medical history forms concerning the viral infection and its symptoms. At-risk patients are identified at the entry and requested to postpone any elective treatments for 14 days, while asymptomatic patients who clear screening are allowed inside with their masks on at all points of time except when receiving treatment [34]. Respirators such as N95 and four-layered masks have been used in conjunction with clear face shields to reduce transmission risk during treatment. After treatment, the clinical area is cleaned, sterilized, and subject to UV lamp disinfection for half an hour. While dentists have been as worried as the public about the infection risks, their experience and added safety approaches have brought about an air of confidence towards infection control.

## 4. Incorporation of Teledentistry

While this modality has been developing slowly in the more developed regions of the world, it has taken time to find its use in the developing areas [35]. It has various benefits to offer such as

1. Improving the oral healthcare delivery to remote areas
2. Making dental care accessible to everyone
3. Bringing down the costs by enabling every person to get regular checkups
4. Helping gather data for research that can be applied to the betterment of oral health
5. Reducing the risk of virus transmission by bringing down physical visits to clinics until the vaccination process is complete

In the Southeast Asian region, many countries such as Thailand and Malaysia have reimposed lockdowns at the time of preparing this review. While this may be effective in reducing the SARS-CoV-2 virus transmission, it is detrimental to oral health as people cannot get access to oral healthcare in certain regions. This situation is where teledentistry has been found to be most effective, as dental practitioners can continue to see their patients virtually. While dentists may not be able to provide most treatment services online, the use of teledentistry has ensured that preventive services such as virtual oral consultations and counseling can be carried out.

### 4.1. Regulations on Teledentistry

As medical regulatory bodies, dental councils of countries in this region have not laid down any specific guidelines for the training, practice, and implementation of teledentistry into daily practice. Teledentistry has been expensive for acquiring the required equipment, and previous studies have reported no revenue for practitioners [36]. Since there is a deficit of established guidelines, there is a marked difference in patient information confidentiality, privacy, liability, and consent regarding its implementation across the Southeast Asian region. There is a distinct lack of measures to maintain control over the safety and efficiency of teledentistry as many users do not have the required training. The Royal College of Dental Surgeons of Ontario is one of the few regulatory authorities that have established guidelines that can be particularly useful for the practice of teledentistry [37]. Salient features of these guidelines include

- The use of audio-video technology for data collection before any medicines are prescribed
- Patient data confidentiality following the guidelines laid down in the Personal Health Information Protection Act, 2004
- Patient data must be accessible only to the dentists and the concerned patients
- Privacy must be maintained during virtual consultations
- Regular monitoring and evaluation of virtual sessions to ensure uniformity in the practice of teledentistry

### 4.2. Types of  Teledentistry

**Figure 1:**
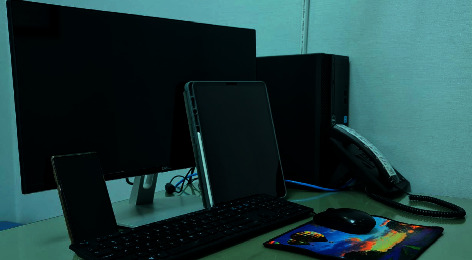
Different screen-based devices used for teledentistry consultations in the ASEAN region.

The American Dental Association has defined teledentistry as the use of telehealth systems and methodologies in dentistry [38]. This modality of providing the patient remote access to healthcare services includes the use of a broad group of technologies, which are categorized as follows:

1. Store and forward: using this medium, a patient's oral health records such as radiographs and photographs can be electronically transferred to a dental practitioner for evaluation. The dentist then analyzes the records and provides a diagnosis via a nonlive medium.
2. Remote patient monitoring: this modality is used for patient data collection from a remote site and then transferring to a dental practitioner in another location. The dental practitioner is then able to provide an evaluation to help treat the patient.
3. Live video: this involves the use of a live interaction between the patient and the dentist utilizing audio-visual communication for diagnosis.
4. Mobile health: it makes use of mobile communication devices such as phones and tablets to provide virtual oral healthcare services or even for educational programs (Figure 1).

The Southeast Asian region during the last decade has undergone a lot of changes with regard to industrialization. Trade and services have seen liberalization that has brought in investments and lifted the economy [4]. Previous studies conducted across the ASEAN (Association of Southeast Asian Nations) region have estimated more than 150 billion dollars of revenue from digital economy-related activities of which connectivity and online services form the two major contributors [39, 40]. This region has also been shown to have among the highest rates of Internet usage globally with Thailand and Indonesia demonstrating double the daily usage compared to the public in the United States [39]. The daily consumption of online media content is also exceedingly high in this region as Vietnam and Thailand have been placed among the top 10 countries for the highest media viewership [39]. The high Internet and online media usage can be utilized for various beneficial purposes, such as the setting up teledentistry centers that could provide oral healthcare access and information to the less-privileged areas.

### 4.3. Challenges

During this period, the main areas of concern have been the availability of personal protective equipment (PPE), transmission risks, and the financial matters arising from reduced patient inflow and the enhanced costs associated with the newer patient screening and sterilization measures [41]. While most dentists in the Southeast Asian region have managed to incorporate most of the guidelines into their daily practice, there is a lingering sense of worry arising from not knowing when the situation would go back to normal. The COVID-19 outbreak has not only brought into focus the occupational hazards associated with the field of dentistry but also has forced us to change and adapt to the new normal [42].

Regarding the implementation of teledentistry and teleorthodontics, there have been various challenges in this region. One of the problems that have been faced in this region is the quality of images required for proper evaluation. Patients can consult virtually, but the quality of diagnosis is severely limited by a lack of appropriate intraoral records such as periapical radiographs [37, 43]. Another crucial element that affects the quality of the virtual session is the time consumed versus the financial returns for the participating dentist. No regulated service provider offers these services in this region to ensure compensation for the dentists for these virtual services [44].

## 5. Future Perspectives

With teledentistry and telemedicine proving to be particularly useful during the pandemic, the World Health Organization (WHO) must set down guidelines that regional regulatory authorities can adopt [45, 46]. Establishing guidelines would aid in solving legal, scientific, and ethical issues occurring with the implementation of transregional programs [47–49]. Guidelines also need to be established regarding human resource training and minimum credentials required to register participating hospitals and clinics. Dentists must be encouraged to join such training as an option and an added standard for dental care.

## 6. Final Considerations

The risk of transmission through dental procedures must be kept to a minimum through precise actions and protocols. Dentists must keep themselves updated with the characteristics of SARS-CoV-2 and the latest infection control measures. As the number of COVID-19 cases continues to increase, dentists must ensure that they participate in advanced infection control programs to strengthen their knowledge [50, 51]. Teledentistry is the clear answer in the coming months as it will help reduce the risk of virus transmission and help patients get access to oral healthcare and dentists to see their patients. This modality needs to be considered by governing and regulatory bodies to ensure a proper structure is followed for best results.

## Figures and Tables

**Table 1:** Association of Southeast Nations (ASEAN) grouped according to the World Bank (Asian human capital index) [3].

Lower middle income	Upper middle income	High income
Indonesia	Thailand	Singapore
Myanmar	Malaysia	Brunei
Philippines		
Cambodia		
Vietnam		
Lao PDR		

**Table 2:** Key economic characteristics of ASEAN countries [4].

Country	Population (in millions)	Per capita gross domestic product (GDP)	Gross domestic product (GDP)
Indonesia	227.75	1.897,57	432,18
Myanmar	57.64	350,14	20,18
Philippines	88.71	1.683,68	149,36
Cambodia	14.32	603,13	8,63
Vietnam	85.15	835,09	71,11
Lao PDR	6.09	693,64	4,22
Thailand	63.03	3.917,89	246,97
Malaysia	27.18	6.872,50	186,83
Singapore	4.83	36.694,53	177,58
Brunei	0.39	31.404,31	12,24

## Data Availability

All data related to the study have been included in the article.
